# Component Model of Addiction Treatment: A Pragmatic Transdiagnostic Treatment Model of Behavioral and Substance Addictions

**DOI:** 10.3389/fpsyt.2018.00406

**Published:** 2018-08-31

**Authors:** Hyoun S. Kim, David C. Hodgins

**Affiliations:** Addictive Behaviours Laboratory, Department of Psychology, University of Calgary, Calgary, AB, Canada

**Keywords:** addictive disorders, treatment, transdiagnostic, substance use disorders, behavioral addictions

## Abstract

Behavioral addictions such as gambling, video games, sex, and shopping share many clinical features with substance use addictions including etiology, course, and neurobiology. Yet, the treatment of behavioral and substance use addictions tends to be separated. However, we argue that a more effective and efficient treatment approach is to conceptualize behavioral and substance use addictions as different expressions of a common underlying disorder and, in treatment, to address the underlying mechanisms common to both. To this end, the article presents a developing transdiagnostic treatment model of addictions that targets underlying similarities between behavioral and substance use addictions, called the component model of addiction treatment (CMAT). The CMAT is transdiagnostic in that it can be used in the treatment of both behavioral and substance use addictions. It is pragmatic in that it targets component vulnerabilities, which are enduring, yet malleable, individual psychological, cognitive, and neurobiological characteristics that are common to all addictive disorders and have been demonstrated to be modifiable. A working model of CMAT is presented, including proposed component vulnerabilities: lack of motivation, urgency, maladaptive expectancies, deficits in self-control, deficits in social support, and compulsivity, as well as their potential intervention possibilities. Future directions and potential implications of the CMAT are discussed.

## Component model of addiction treatment: a pragmatic transdiagnostic treatment model of behavioral and substance addictions

The publication of the Diagnostic and Statistical Manual of Mental Disorders (DSM-5) (1) marked a significant shift in the field of addictive disorders. For the first time in history, a behavior, as opposed to a psychoactive substance was classified as an addiction. Specifically, gambling disorder (previously called pathological gambling) was moved from the Impulse Control Disorders section of the DSM to the Substance Related and Addictive Disorders section. The re-classification of gambling disorder occurred due to decades of accumulating evidence that gambling disorder shares many commonalities with substance use disorders, which have been well elucidated and summarized in the existing literature (2).

In addition to gambling, internet gaming disorder (i.e., video game addiction) is currently listed in section Unified Theories of Addictive Disorders of the DSM-5 Emerging Measures and Models as a potential psychiatric disorder pending further empirical investigation. Likewise, gaming disorder is included alongside gambling and substance use disorders in the upcoming edition of the International Statistical Classification of Diseases and Related Health Problems−11th Revision (ICD-11) (3). The inclusion of gaming disorder in the DSM-5 and ICD-11 stems from empirical research delineating the similarities between gaming disorder and gambling (4), as well as substance use disorders (2). The inclusion of the aforementioned behavioral addictions in the manual of psychiatric disorders speaks to the rising relevance of behavioral addictions in both research and treatment in the fields of psychology and psychiatry.

## Behavioral and substance addictions: two sides of the same coin?

The past several decades have seen a remarkable growth in the research of behavioral addictions (2). Similarly to gambling and internet gaming, empirical research has examined other compulsive behaviors which have been postulated as behavioral addictions. These behaviors include, but are not limited to: compulsive buying, sex addiction, binge eating, work addiction, exercise addiction, and smartphone addiction (2, 5–8). The overlapping feature common to all behavioral addictions is the failure to resist an impulse or urge, leading to persistent engagement in the behavior (e.g., video games, shopping) despite recurring harms (2).

Despite the similarities between behavioral addictions and substance addictions, there is a debate in the empirical literature as to whether behavioral addictions should be classified as “new” psychiatric disorders (9). Furthermore, there has been a trend in the “scope creep” of behavioral addictions, whereby an increasing number of everyday activities have been proposed as addictive disorders, including for example tanning addiction (10), tango addiction (11), and fortune telling addiction (12). However, what is remarkable when examining the relationship between addictive disorders including both behavioral and substance addictions is the similarities rather than the differences. Indeed, there is considerable overlap in etiological (e.g., onset, natural course), phenomenological (e.g., cravings, pre-occupations), and clinical (e.g., treatment strategies, co-morbidities) presentations across addictive behaviors (2). For instance, behavioral addictions such as gambling and internet gaming disorder, much like substance use disorders, tend to have their onset in late teens or early twenties and follow a variable course of lapses and recoveries (13, 14). Behavioral and substance addictions also tend to share similar risk factors. Adverse childhood experience or childhood trauma such as physical and emotional abuse have been linked to increased risk of developing a variety of addictive disorders including addiction to alcohol, gambling, video games, shopping, and sex (15). In addition, dysregulation in underlying neurobiology such as the dopamine reward system has been found in problematic engagement with gambling (16), video games (17), and shopping (18), and both behavioral and substance addictions share similar executive functioning deficits as demonstrated by deficits in decision making and difficulties in delaying rewards (2).

Importantly, the considerable overlap shared across addictive disorders may have potential treatment implications. Specifically, both behavioral and substance addictions share common clinical processes that may be targeted in treatment. For example, impulsivity, the tendency to act rashly without forethought, has been found to be a key characteristic in a wide array of behavioral addictions including gambling (19), video games (20), sex (21), and shopping (22). Compulsivity is present in both behavioral (23) and substance addictions (24). Emotional dysregulation or low distress tolerance has been associated with gambling (25), compulsive shopping (26), and binge eating (27) and may increase the severity of the addictive behaviors (28). Lack of social supports and interpersonal conflicts have also been demonstrated to negatively affect the onset and severity of substance use disorders such as alcohol (29) and a variety of behavioral addictions (30, 31).

Although there are similarities between behavioral and substance addictions, there are also important neurological differences. For instance, whereas the role of neurotransmitters, specifically dopamine, is robustly implicated in substance use disorder, especially stimulants, the role of neurotransmitters is less clear when it comes to behavioral addictions such as gambling (32). Indeed, a recent meta-analysis of 25 studies on reward processing found increased activation in the ventral striatum during reward outcomes for substance use disorders, whereas gambling addiction was associated with decreased activation in the dorsal striatum (33). Neurological differences have also been found in internet gaming disorder. Compared to alcohol use disorder, internet gaming disorder has been associated with stronger functional connectivity in the left ventromedial prefrontal cortex (34). That said, what is known is that engaging in both behavioral and substance addictions results in the activation of the dopamine reward system, with continued engagement being associated with structural and functional changes (2). In these ways, behavioral addictions closely mimic the hallmark characteristics of substance use disorders (35).

## Unified theories of addictive disorders

The similarities among addictive disorders, including behavioral addictions have been noted for decades. Indeed, theoretical models of addictive disorders that view addictions as a common disorder rather than distinct disorders have been proposed as early as in the 1980s (36). The general theory of addictions by Jacobs (36) placed emphasis on two predisposing factors that make an individual at risk for developing an addiction: (i) chronic hypo or hyperarousal and (ii) maladaptive schemas of oneself as inferior. Jacobs (36) argued that coping with negative emotions by engaging in an addictive behavior is a key maintaining factor of addictions. In addition, Jacobs (36) delineated a process model of addictions, which includes three phases: (i) Phase I, the initial discovery in which one learns that engagement in addictive behaviors can alleviate negative affect, (ii) Phase II, the phase in which the positive reinforcing effects of the addictive behavior become over-learned and lead to compulsive-like behaviors and are thus resistant to change, and (iii) Phase III, the phase in which the individual actively avoids experiencing the aversive state that the addictive behavior was alleviating by continuing to engage in the addictive behavior despite the continued harms. Jacobs (36) argued that the predisposing factors and the three phases are uniform across all addictive behaviors.

Orford (37, 38), in his excessive appetites model of addictions, emphasized psychological processes that lead to an appetitive behavior such as alcohol use, smoking, gambling, drug use, eating, and sex that may become excessive. Orford's highlighting of psychological processes was a significant contribution given many theories of addictions focused on the physiological processes that result from ingestion of a psychoactive substance. The focus on psychological processes acknowledges a range of activities that may lead to impairments with excessive engagement. In other words, this theory provides a conceptual model of addictions that allows for the inclusion of behavioral addictions. The excessive appetites theory of addictions shares overlapping components with the general theory model of addiction, including learning processes in which people associate addictive behavior with alleviation of negative affect (i.e., emotional regulation).

The syndrome model of addiction (39) introduced the concept of multiple and interacting biopsychosocial antecedents, manifestations, and consequents of addictive disorders. Shaffer et al. (39) described the addiction syndrome as a cluster of signs and symptoms related to a common underlying dysfunction. The presence of a syndrome suggests commonalities between different expressions of addictive behaviors, and these commonalities share similar etiologies. The environment, which allows repeated interactions with a specific substance or behavior, determines the specific addiction. An important contribution of the syndrome model of addiction is the acknowledgment that there are, as well, unique features associated with each specific addictive behavior, despite the underlying syndrome. For example, if a person repeatedly engages in alcohol use, then the manifestation of the addiction syndrome and its consequences will have some characteristics that uniquely reflect problems associated with alcohol such as high blood pressure, liver cirrhosis, and pancreatitis. Conversely, if one interacts repeatedly with a slot machine, then the manifestation of this syndrome will have some features that uniquely reflect gambling such as chasing losses and financial debt. Internet gaming may lead to sleep disturbances such as insomnia given the significant amount of time an individual can spend playing video games (40). However, the various expressions of addiction will also share common manifestations and sequelae such as psychological distress, the use of addictive behavior to cope with negative affect and impairments in family life, and work life.

The components model of addiction also conceptualizes addictive disorders based on their commonalities (41). According to this model, all addictive behaviors consists of six core components: (i) salience, which refers to the addictive behavior becoming the most important activity in a person's life and may manifest as pre-occupation or craving; (ii) mood modification which refers to subjective enhancement such as getting high or alleviating negative affect, in other words, coping; (iii) tolerance which is the need to increase the frequency, duration, or amount of a particular addictive behavior to get the same effects; (iv) withdrawal symptoms, which are unpleasant physiological and psychological effects experienced when an addictive behavior is discontinued; (v) conflict that can be either personal and interpersonal that arise due to continued engagement in addictive behaviors; and (vi) relapse, which refers to the reversion back to previous levels of engagement when attempting to reduce an addictive behavior. Griffiths (41) argued that for a behavior or substance to be conceptualized as an addiction, all of the above components need to be demonstrated.

The above models are similar in that they each postulate, in one fashion or another, the commonalities between addictions. However, there are also important differences. The syndrome model of addictions (39) acknowledges that despite the similarities across addictions, there also exists unique manifestations. Orford privileged certain addictions (e.g., alcohol, nicotine, gambling, food, and sex) as “excessive appetites” whereas the other models remain relatively impartial, with the exception of noting that alcohol and gambling are the prototypical substance and behavioral addiction respectively. Additionally, each model presents strengths and weaknesses. For example, the general theory of addictions (36) was the first to propose a unified theory of addictions. However, the model was based on gambling, and thus was unable to take into account the proliferation of behavioral addictions that exists today. A strength of the excessive appetites model (38) is expanding the scope of behavioral addictions to include food and sex. A potential weakness of this model is a minimal focus on physiological processes of addictions. An important contribution and strength of the syndrome model of addictions (39) is introducing the concept of unique manifestations in addictions. Lastly, a strength of the components model of addictions (41) is providing a model that reduces the similarities of addictions to six core components. However, a potential weakness of such a parsimonious model is the exclusion of other components, which may be important characteristics of both behavioral and substance addictions (e.g., compulsivity).

## All for one or one for all? toward a transdiagnostic treatment of addictions

The aforementioned theories have all alluded to the potential treatment implications of viewing addictive behaviors as a common underlying disorder. Yet, a unified transdiagnostic treatment model for addictive disorders has not emerged. In contrast, the trend over the past number of decades in the development of evidence-based treatments for addictive disorders as well as other mental health disorders has been the development of single diagnosis protocols. Indeed, disorder-specific protocols are readily available for both substance and behavioral addictions (42, 43). That said, the diversity in treatment programs are likely the result of responding to the needs of clients, whereas the training of clinicians likely impacts the management of different disorders.

Although protocols have not been developed that capitalize on common underlying factors for addictions, clinicians often intuitively target the underlying similarities in the treatment of their clients' addictions, regardless of whether the presenting problem is alcohol, cannabis, gambling, or sex. Indeed, it has been argued that due to increased demand for treatment, the field of addictions treatment has out of necessity, utilized a more holistic approach and has applied a broader focus on examining processes that underlie multiple problem areas (44). Providing support for this supposition, evidenced-based treatments for addictions such as cognitive behavioral therapy (CBT) for substance use disorders (44, 45) and motivational enhancement therapies (46) use the same treatment strategies regardless of the specific substance or behaviors. In addition, there exists a multitude of 12-step programs for distinct addictive behaviors such as alcohol (Alcoholics Anonymous), cocaine (Cocaine Anonymous), gambling (Gamblers Anonymous), sex (Sexaholics Anonymous), and eating (Overeaters Anonymous). 12-step programs largely operate independently and are disorder-specific, emphasizing each groups' need to embrace “singularity of purpose.” That is, an individual who is experiencing problems with alcohol will attend Alcoholics Anonymous, whereas an individual experiencing problems with gambling will attend Gamblers Anonymous and individuals experiencing both disorders are encouraged to attend both groups. However, regardless of which 12-step program an individual attends, the principles of the program and the 12-steps remain very similar. Implicitly then, the treatment of addictions may closely resemble a transdiagnostic approach in practice.

To summarize, there exists a considerable overlap between behavioral and substance addictions, including in psychological processes that may be targeted in treatment. In this light, we present a developing transdiagnostic treatment model for addictions that takes advantage of the underlying commonalities that have been shown to be amiable to change across both behavioral and substance use addictions.

## Transdiagnostic treatments

The term transdiagnostic treatment is used variably to describe a number of different approaches to providing treatment. Sauer-Zavala et al. (47) recently distinguished among three broad categories, all of which have empirical support for their efficacy. The first of these are universally applied therapeutic principles. Treatments such as psychodynamic and CBT models are transdiagnostic in the sense that they are designed to be applied to a variety of presenting conditions. Included in this category are mindfulness-based interventions and acceptance and commitment therapy (ACT) (48). The second type of transdiagnostic treatments are modular treatments that provide clinicians with a number of evidence-based treatment strategies that can be applied according to individualized patient needs. The Harvard University's modular approach to therapy for children with anxiety, depression, or conduct problems (MATCH) (49) is a well-regarded example. The third type of transdiagnostic treatments are interventions that specifically target shared mechanisms that have been implicated in the etiology or maintenance of a group of disorders. These models target what are presumed to be core features of groups of disorders, such as avoidance coping related to high neuroticism, which is targeted by the Unified Protocol for transdiagnostic treatment of emotional disorders (50), and preoccupation with body weight and shape, which is targeted by Fairburn's Enhanced CBT model for eating disorders (51).

Emerging research suggests that transdiagnostic treatments lead to superior outcomes when compared to control conditions and treatment as usual. A meta-analysis of 24 randomized control trials for transdiagnostic treatments for anxiety and depression found medium to large effect sizes in favor of transdiagnostic treatments compared to no treatment control conditions, such as waitlists, and small but significant effect sizes when compared to disorder-specific treatments such as treatment for social anxiety. It has been argued that a benefit of transdiagnostic treatments is that they treat not only the presenting problem, but can also concurrently treat co-occurring problems (47). For example, transdiagnostic treatments for anxiety disorders have demonstrated modest but significant improvements in symptoms of depression, without explicitly treating the depressive disorder itself (50). This is an immense benefit of transdiagnostic treatments in that co-morbidity is the rule rather than the exception in psychiatric disorders (52), including addictive disorders (53).

Applied to the treatment of addictions, rather than targeting a specific addictive behavior, which is the traditional treatment approach, it may be possible to simultaneously influence a variety of current and emerging addictive behaviors by targeting common underlying mechanisms (i.e., component vulnerabilities). This flexible approach benefits not only “traditional” addictions such as gambling disorder or substance abuse, but uncommon and underserved behavioral addictions such as video games, compulsive shopping, sex addiction, and others, which clinicians who specialize in substance use addictions might not feel competent in being able to treat (6). Herein, we define component vulnerabilities as enduring yet malleable individual characteristics that are linked to different expressions of addictive disorders. Some of these vulnerabilities include lack of motivation, the disposition to act rashly when experiencing strong emotions, deficits in self-control, expectancies, and motivations for engagement in addictions, family and social support deficits, executive functioning deficits, and compulsivity.

## Potential benefits of transdiagnostic treatment for addictions

Similar to transdiagnostic treatments for other psychiatric disorders such as anxiety (50), a transdiagnostic treatment approach to addictive disorders would have several benefits compared to the current treatment model of targeting specific addictions. First, treatment would be more efficient. This is because both behavioral and substance addictions are highly co-morbid with one another. For instance, gambling disorder frequently co-occurs with substance use disorders, with point prevalence rates of 58% for any substance use disorder (54). Similarly, previous research has found that substance use disorders co-occur up to 38% with internet use disorder, 46% with compulsive buying, and 64% with sex addiction (2). Additionally, in a large representative sample of Canadian adults, 40% of participants who reported experiencing problems with an addictive behavior in the past 12 months, reported problems with two or more substance or behavioral addictions, with high co-occurrence of both substance and behavioral addictions in individuals (6).

It has long been speculated that the reason for the high degree of co-occurrence of addictive disorder is due to the underlying psychological mechanisms that link two addictive disorders (47). Recent research provides empirical support for this assertion. For example, negative urgency, which is the tendency act rashly under intense negative affect has been suggested to be an important construct that underlies the co-occurrence of gambling and nicotine use (55) as well as alcohol use disorder (56). Thus, rather than taking a sequential approach to treatment by treating first the substance use disorder and then the behavioral addiction or vice versa, the treatment of the co-occurring addictions can proceed in an integrated fashion by targeting the underlying component vulnerability. In the above example, it is possible to influence the alcohol, gambling, and nicotine use by targeting negative urgency, the underlying mechanism that is leading to the expression of all three addictions. Targeting component vulnerabilities in treatment is likely to lead to improvements in not only the primary addiction but also in any secondary addictions that may be present.

The second benefit of transdiagnostic treatments is cost-efficiency (57). There currently exists a variety of treatments for addictive disorders, including for example, psychoanalytic, narrative therapy, solution-focused brief therapies, cognitive behavioral therapy, acceptance-based commitment therapies, and motivational enhancement therapies among others (2, 44, 58–60). In addition to this, several unique therapies have been developed for specific behavioral addictions (42, 61). It is virtually impossible for clinicians to learn and become competent in delivering the dozens of treatment approaches that currently exist or learn new treatments for specific addictive disorders, let alone the unwieldy training costs. A more fruitful approach is to train clinicians in a unified treatment approach that can be used for both behavioral and substance addictions. Indeed, while many treatment models for addictions exists, there is a high degree of overlap in the mechanisms that lead to treatment outcomes (39). Relatedly, the ability for clinicians to treat both behavioral and substance use addictions will allow current services to expand their scope of practice versus creating new services for emerging behavioral addictions.

This approach is in contrast to the current model of treatment services for behavioral addictions. For example, with the expansion of legalized gambling in the 1990s, many jurisdictions funded gambling-specific treatment services that were administratively separate from substance abuse services. A similar trend is beginning to occur for behavioral addictions, where specialized treatment programs are starting to be developed (62). Unfortunately, the creation of specific treatment centers for behavioral addictions may have several unintended consequences, including perpetuating the idea that behavioral addictions represent “unique” psychiatric disorders, despite the empirical literature highlighting considerable overlap between behavioral and substance addictions (2). Clinically, the creation of specific treatment centers maintains the separation of treatment of behavioral and substance addictions, which can be problematic as individuals experiencing problems with emerging addictive disorders, including video gaming, may not be able to access the services they need. Further, for individuals with multiple addictive disorders, it may present confusion as to which treatment services to seek and may result in multiple referrals.

A third benefit to targeting underlying commonalities is that it may decrease the likelihood of individuals engaging in a concept known as addiction substitution. Addiction substitution occurs when an individual who recovers from one addictive behavior (e.g., alcohol) then substitutes their dependency to another addiction (e.g., gambling). Although the empirical literature on addiction substitution is sparse, what is known is that there is considerable change, both increases, and decreases, in other addictive behaviors during recovery. For example, in a large national representative sample of adults from the United States, Blanco et al. (63) found that 13% of people who recovered from a substance use disorder at Time 1, reported having developed a new onset of substance use disorder at Time 2. Furthermore, Hodgins et al. (64) found that among recovered cannabis users, only a small minority (14%) reported no change in other addictive behaviors. Indeed, most people reported that their addictive behaviors either increased (26%), decreased (39%), or both increased and decreased (21%). Interestingly, treatment seeking cannabis users were more likely to report decreasing other substance use and less likely to report an increase in other substance use upon recovery compared to cannabis user who utilized self-directed change. A potential reason for these findings may be due to the fact that in treatment, it is more likely that some aspects of the component vulnerabilities will be addressed as compared to self-directed change, in which the focus may be on the specific addictive behavior.

## Candidate component vulnerabilities (transdiagnostic mechanisms)

**Figure 1:**
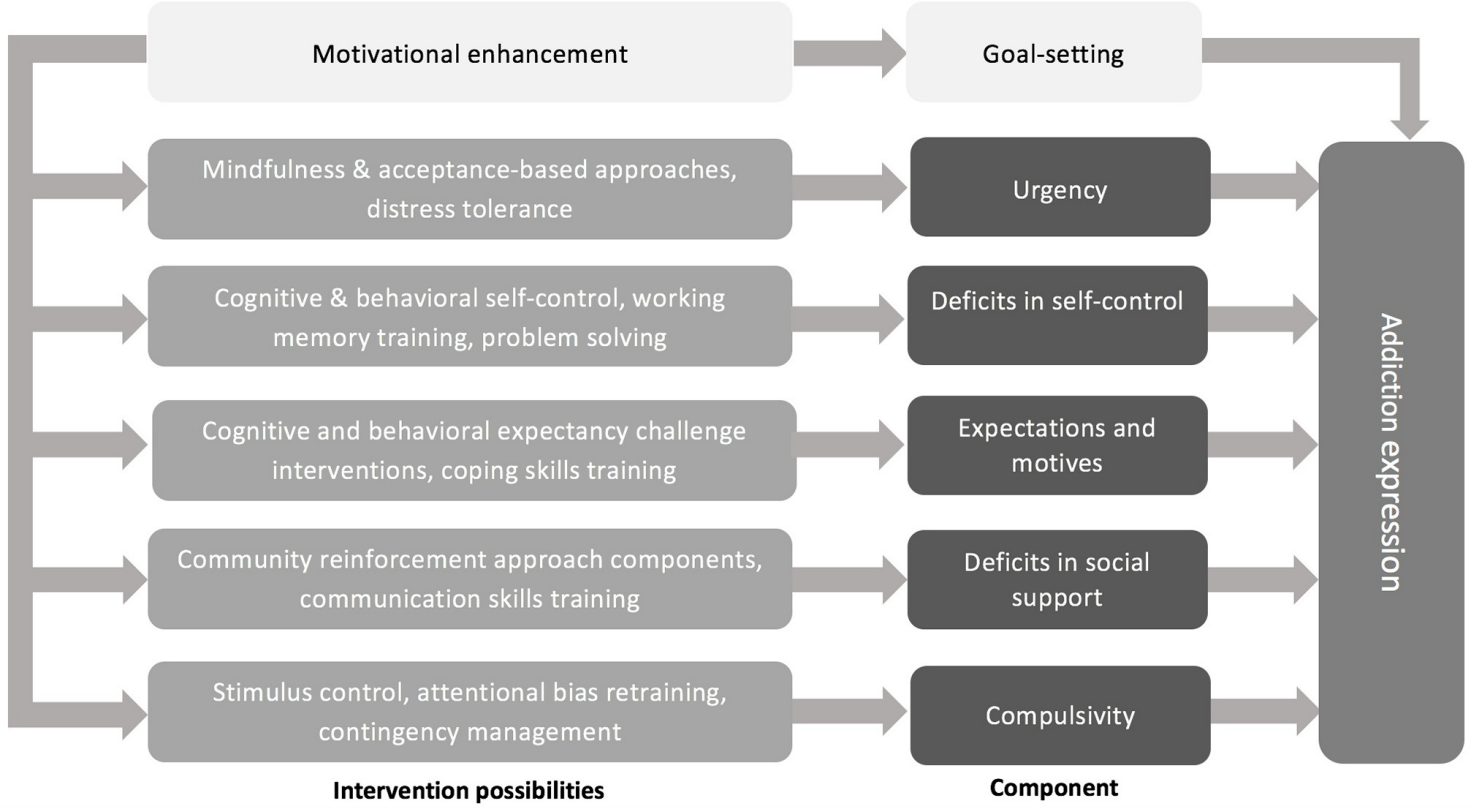
A working model of the component model of addictions treatment depicting component vulnerabilities and the corresponding intervention possibilities.

Harvey et al. (65) have distinguished between transdiagnostic factors that are descriptively transdiagnostic and those that are mechanistically transdiagnostic. Descriptively transdiagnostic factors are those that are present across disorders but are not etiological or maintaining conditions. Mechanistically transdiagnostic factors are those shown to be causally linked to multiple disorders. In our pragmatic model we include both types of these factors as both can be can targeted in treatment. Over time, empirical evidence will reveal which vulnerabilities are, in fact, important treatment targets. Figure 1 outlines our developing component model of addiction treatment, highlighting the component vulnerabilities and the corresponding intervention possibilities. With the exception of motivation, the model does not assume a temporal sequencing of the component vulnerabilities to be addressed in treatment. Rather, the clinical decision of sequencing would be determined by individual client needs, specifically assessing which of the component vulnerabilities are most likely to lead to, and maintain the expression of the addictive disorder for each client.

Our pragmatic list of vulnerabilities is not meant to be comprehensive, but it is representative of psychological processes to date that, although enduring, are amenable to change. Furthermore, while we acknowledge that evidence supports high genetic liability among addictions (66), the genetic level of analysis is not currently easily translated into personalized treatment and thus we have omitted the genetic component vulnerability from the model. In contrast, our treatment model focuses on component vulnerabilities that can be directly targeted in treatment and have been shown to be important processes in the expression of both behavioral and substance addictions. In our opinion, we view the component vulnerabilities as culturally invariant because they represent psychological processes that are innate human conditions. While we acknowledge that in certain cultures, a candidate component vulnerability may be more or less likely to play a central role in the expression of an addictive behavior, in all cultures we would expect all the component vulnerabilities listed below to exist to varying degrees.

## Deficits in motivation for change

A cardinal characteristic of addiction is the failure to engage in change despite knowledge that recovery is indeed feasible and, furthermore, that changing the pattern of the addictive behavior is in the best interest of the individual (67). In fact, DiClemente stated that change is the antithesis of addiction (67). While most people do make an attempt to change their addictive behaviors, it typically occurs after several years, and with the accumulation of negative consequences (68, 69). Providing support for this assertion, only 15% of people are actively engaged in removing a problematic behavior from their behavioral repertoire (70). Indeed, ambivalence about change is common among people who engage in a wide array of addictive behaviors including behavioral addictions (71). Unfortunately, however, low motivation has been shown to predict poorer treatment outcomes, including premature treatment termination (72). Thus, prior to addressing the component vulnerabilities listed below, we argue that motivation to engage in change must be addressed if treatment is to be successful and, as such, we consider motivation to change to be an overarching component vulnerability in Figure 1.

Motivational enhancement therapies (MET) (46), including motivational interviewing (MI) (70) represent a potential intervention possibility in helping to increase the motivation of clients in overcoming their addictive disorders and to engage in the required treatment processes. MET is a type of client-centered therapy that helps to address ambivalence and enhance an individual's internal motivation to engage in behavioral change. MET assumes that motivation is dynamic and that people are at different stages of motivation when it comes to overcoming addictive behaviors. The goal of MET is to evoke change talk in a supportive and collaborative environment and foster the client's own internal motivation to engage in change (44). Decades of empirical evidence, including randomized controlled trials, support the use of MET in the treatment of addictive disorders. Several meta-analyses have found that MET is associated with significantly improved outcomes when compared to non-treatment controls and is equivalent to other treatments (73). Specifically, motivational enhancement and interviewing have been supported in the use of a wide variety of addictive behaviors, including alcohol, tobacco, marijuana, and gambling (74). In addition, a recent meta-analysis found that pre-treatment motivational interviewing was associated with greater treatment engagement compared to comparison groups in a variety of mental health settings (75).

The potential benefits of enhancing motivation to improve treatment outcomes for addictive behaviors have also been noted by incorporating motivation enhancement as an adjunct to other treatments (76). McKee et al. (77) found that although there were no differences between participants who received one session of motivational enhancement therapy combined with three sessions of cognitive behavioral therapy compared to those who only received the cognitive behavioral therapy in reduced cocaine use, the inclusion of motivational interviewing resulted in greater treatment attendance following the treatment intervention as well as greater desire for abstinence. In a sample of substance users who also presented with co-occurring gambling problems, Petry et al. (78) found that a combination of motivational enhancement and cognitive behavioral therapy led to greater improvements in gambling outcomes than a brief single session intervention. Motivational interviewing has also been combined with acceptance and commitment therapy with promising results (79).

In addition to helping to resolve ambivalence and enhancing motivation to change, MET can also help clarify and specify individuals' goals for change. Having adaptive and realistic goals is important in facilitating effective self-control of actions and emotions. Moreover, in addictive disorders, establishing clear goals to moderate or cease addictive involvement is an important part of treatment.

## Negative urgency

Urgency is the disposition to act in rash, ill-advised ways when experiencing intense positive (positive urgency) or negative (negative urgency) affect (80). Negative urgency is the integration of negative affect and impulsivity. In this way, negative urgency helps to explain why people engage in impulsive actions, for example addictive behaviors when emotionally dysregulated (81). Urgency is one of the facets of impulsivity that has received increasing empirical attention given its hypothesized association in the etiology and maintenance of addictions. Negative urgency, in particular, has been robustly associated with substance use disorders such as alcohol (82) as well as behavioral addictions such as gambling (83), video games (84), compulsive buying (85), binge eating (86), and sex (87).

In a meta-analysis of 96 studies (*N* = 32,167) examining the relationship between facets of impulsivity and its association with problematic alcohol use, Coskupinar et al. (82) found that while impulsivity, in general, was related to alcohol use, negative urgency was the only facet of impulsivity that predicted alcohol dependence and drinking-related problems. The association between negative urgency has also been documented in longitudinal studies with behavioral addictions. In a large sample of Canadian adults (*N* = 1,002), Farstad et al. (86) found that negative urgency was the only facet of impulsivity that predicted problematic gambling and binge eating, suggesting negative urgency may be an important transdiagnostic mechanism in the expression of both gambling disorder and binge eating disorder, and a component of impulsivity that needs to be addressed in treatment. Indeed, negative urgency has been related to poorer treatment outcomes including relapse in substance use disorder (88). Furthermore, a meta-analysis examining changes in facets of impulsivity reported that sensation seeking and negative urgency were the only facets of impulsivity that significantly decreased during treatment (88).

Distress tolerance is a construct that is related to urgency in that it reflects perceived and actual capability to withstand negative emotional or physical states. Lower distress tolerance, as assessed with simple behavioral tasks (e.g., breath holding, hand grip persistence), also predicts negative treatment outcomes, including increased relapse across substance abuse, smoking, and gambling disorder (89). In addition, distress intolerance amplifies the distress-terminating effects of addictive behaviors (90). Thus, the inability to tolerate negative emotions appears to be an important factor in the etiology and maintenance of both substance and behavioral addictions.

Distress tolerance can be targeted in skills and exposure-based treatments, in which individuals practice cognitive and behavioral tolerance techniques in the context of negative affect. Bornovalova et al. (91) provide preliminary evidence that a six session Skills for Improving Distress Intolerance Program added to residential substance use disorder treatment showed both improvement in distress tolerance skills and clinical outcomes compared with supportive counseling. Furthermore, among smokers with a history of early relapses, people who were randomly assigned to a distress tolerance treatment were over six times more likely to be abstinent compared to a standard smoking cessation treatment, with the effects being maintained, albeit diminishing overtime (92). More recently, Stein et al. (93) assessed the effects of including a distress tolerance intervention to buprenorphine in the treatment of opioid dependence. At 3 months, 36.5% of people randomly assigned to the distress tolerance program were opioid negative compared to 28% of people who were randomly assigned to the health education program. Although not statistically significant, the distress tolerance intervention led to a small reduction in opioid use. In sum, the literature provides promising support for targeting distress tolerance in the treatment of addictive behaviors.

A recent advancement in the treatment of psychiatric disorders has been the emergence of a body of empirical literature supporting the use of mindfulness-based therapies in the treatment of psychiatric disorders including addictive disorders (94). Mindfulness is broadly defined as attending to the present moment in a non-judgmental manner and reaching a state of awareness that can be cultivated through formal and informal practice (95). Mindfulness is included as a component in dialectical behavior therapy and acceptance and commitment therapy as a technique to promote non-judgmental acceptance of internal physiological, cognitive, and emotional experiences (44). Similarly, mindfulness-based cognitive therapy [MBCT; (79)] is provided to reduce the likelihood of relapse into major depression by encouraging observation versus reaction to negative cognitions. Recently, mindfulness based interventions have been developed in the treatment of addictive disorders (e.g., Mindfulness Based Relapse Prevention) with promising results (96). A systematic review concluded that mindfulness-based interventions have demonstrated support for reducing severity of a wide variety of addictions, including behavioral addictions (97). Interestingly, the authors found that combining mindfulness-based interventions with other active treatments led to the greatest efficacy, suggesting the importance of mindfulness as an intervention strategy in addictive behaviors.

In practice, mindfulness-based interventions help individuals become aware of their specific triggers and increase an individual's ability to stay in the moment with discomforting states (96). In this way, cultivating mindfulness may help individuals become less behaviorally reactive when experiencing negative affect. Indeed, it has been found that a mechanism by which mindfulness helps improve mental health functioning is through lessening cognitive and emotional reactivity (91, 98). Although empirical studies testing the mechanisms by which mindfulness-based interventions lead to improved outcomes for addictive disorders are sparse, there is preliminary support that one potential mechanism is reduction of negative urgency. A review of mindfulness-based interventions for substance use concluded that mindfulness meditation enhances peoples' emotion regulation skills, which is a component of urgency as well as reducing drug use (99). Additionally, in a sample of smokers, Spears et al. (100) found that greater self-efficacy for managing negative affect without turning to nicotine use was a mechanism by which mindfulness-based interventions led to improved outcomes compared to usual care. Thus, there is increasing support for urgency as a potential intervention target in the treatment of addictive disorders.

## Deficits in self-control

Self-control refers to the ability to focus awareness beyond immediate stimuli (101). It involves the ability to purposely direct one's actions toward a goal (102), which may involve short terms goals such as limiting the time spent playing video games, not stopping at the bar on the way home, as well as long terms goals such as abstaining from an addictive behavior. This vulnerability has been examined in various research lines showing that addicted individuals have significant deficits, including shortened time perspective and self-control resource depletion. It has been suggested that people have a finite amount of self-control capacity, known as the resource depletion model, whereby if individuals use their self-control capacity for multiple tasks, less becomes available for other tasks (103).

A related construct to deficits in self-control is deficits in executive functioning. Executive functioning are cognitive functions that direct the ability to organize, plan, problem-solve, and coordinate thought and action toward goal-directed behavior, thus facilitating self-control (104). It consists of several top-down cognitive processes such as inhibitory control and working memory (105). It is now well established that deficits in executive functions measured by tasks such as the Iowa Gambling Task and Wisconsin Card Sorting Task have been implicated in a wide array of addictions including both substance use disorders (2, 106) and behavioral addictions (107, 108).

There are several intervention possibilities to increase individuals' self-control capacity, including working memory training to improve executive functions (109). Several empirical studies support the use of working memory training in the treatment of addictions. Houben et al. (110) randomly assigned problem drinkers to 25 sessions of working memory training or control tasks. They found that participants who completed the working memory training showed improvements not only in working memory but reduced alcohol intake at 1 month follow up. Importantly, the reduction in problematic drinking was mediated through improvements in working memory. Additionally, preliminary evidence suggests that computerized tasks such as the Dual N-Back task can enhance executive functions and may show promise in the treatment of addictions (111).

Self-control training has also been shown to improve self-control and help with smoking cessation (112). There are a variety of tasks to improve self-control ranging from strengthening one's hand grip to avoiding sweets and implementation intentions, which are if-then statements created to help with high-risk situations (113). There is now empirical evidence to support that self-control capacity can be enhanced through deliberate practice (103). Goal management training (114), designed to remediate executive dysfunction, has been shown to be effective in improving response inhibition and decision-making in individuals with alcohol problems (115).

Problem-solving therapy is another transdiagnostic approach to addressing deficits in self-control that impede effective problem resolution (116). A central component of this approach is training individuals to use a structured process to identify possible solutions to well-defined problems to combat cognitive and emotional overload, biased cognitive processing of emotion-related information, and ineffective problem-solving strategies (116). Although problem-solving therapy has demonstrated empirical support for the treatment of other psychiatric disorders, specifically depression (117) and is included as a treatment intervention in some treatment manuals for addictive disorder (43), to our knowledge, no studies have directly tested the potential of problem solving therapy in the treatment of addictions.

## Expectancies and motives

Cognitive expectancies for the effects of addictive behaviors have been found to be an etiological and maintaining factor of addictive disorders (118). To this end, two types of dysfunctional beliefs have been identified: permissive beliefs and anticipatory beliefs (45). Permissive beliefs are thoughts that provide a justification for engaging in addictive behaviors, for example, “*it has been a long week; I deserve this.”* On the other hand, anticipatory beliefs are thoughts in regard to what engaging in addictive behavior will do for the individual, such as “*drinking will help me feel better.”* Both types of beliefs may serve to maintain and exacerbate engagement in addictive behaviors. CBT has been identified as the gold standard treatment for a variety of substance use disorders (119, 120), including behavioral addictions such as gambling (121). A component of CBT is helping individuals identify and challenge maladaptive cognitions that are maintaining the addictive behavior (44). Specific cognitive and behavioral substance use expectancy challenge interventions have also shown efficacy (122, 123). For example, a meta-analysis involving 14 studies with 1,415 participants found that compared to control conditions, expectancy challenge interventions resulted in reducing positive expectancies in regard to alcohol. Importantly, expectancy challenge interventions also resulted in improved treatment outcomes for problem drinking (122). Restructuring of maladaptive cognitions have also demonstrated efficacy as a treatment target for gambling disorder (124).

Relatedly, motives for why people engage in addictive behaviors have been prospectively linked to problematic engagement in a variety of addictive disorders (57, 125). Generally, speaking, three primary motives for engaging in addictive disorders have been identified. These motives include: (i) enhancement motives (i.e., engaging in addictive behaviors to enhance excitement and positive affect), (ii) social motives (i.e., engaging in addictive behaviors for social benefit), and (iii) coping motives (i.e., engaging in addictive behaviors to alleviate negative affect). The empirical literature has consistently found that of all the motives, coping motives has been robustly associated with problematic engagement of addictive behaviors, including both behavioral and substance addictions (56, 126). Moreover, our recent work suggests that common motives underlie comorbid alcohol, gambling, and eating problems (127, 128).

Coping skills training is based on the premise that people engage in addictive behaviors to alleviate negative affect (129). If an individual's only means of coping is to engage in addictive behaviors, then an effective treatment strategy would be to help individuals develop more adaptive ways of coping. Adaptive coping skills can vary widely from practicing intrapersonal skills including relaxation training to interpersonal skills such as practicing refusal skills. Coping skills training has been shown to lead to greater treatment improvements as an adjunctive therapy (130, 131). In a sample of marijuana users, Litt et al. (131), tested the mechanisms of behavior change in one of four conditions; control, motivational enhancement plus coping skills, contingency management, and combination of all three active treatments. The results found that longer term abstinence of marijuana was predicted most strongly by the use of coping skills. Coping skills training has also been demonstrated to reduce problem drinking up to 12 months post treatment (130). Moreover, in individuals addicted to gambling, Petry et al. (132) found that regardless of whether problem gamblers were randomly assigned to attend a self-help group or self-help plus professional treatment, coping skills increased over time, although those who received professional treatment reported greater increases in coping skills. Importantly, increased coping skills partially mediated improved treatment outcomes at 2-month post treatment.

## Deficits in social support

Deficits in social support have been consistently linked to the expression of addictive disorders, including alcohol (29), cannabis (133), illicit drugs (134), as well as behavioral addictions such as gambling (135) and video games (31). Furthermore, lack of social support has been associated with poorer treatment outcomes (136), and increases the chance of relapse (137). For instance, interpersonal conflicts may result in increases in negative affect, which then leads an individual to engage in addictive behaviors as a means of coping (138). Enabling, that is, the well-intentioned but unhelpful behaviors of friends or family is another concept that has been shown to increase the use of addictive behaviors (139).

Interventions that enhance and reinforce social and family supports are well supported in the treatment of addictive disorders (138). For example, a therapeutic benefit offered by 12-step programs is social support such as access to a sponsor (140). Additionally, family-based therapies and behavioral couples therapy have shown efficacy in the treatment of a variety of addictive disorders (141–143). An approach that has garnered increasing support in the treatment of addictions is the community reinforcement and family training (CRAFT) approach. The CRAFT approach, involves including concerned significant others of addicted individuals in treatment to engage the addicted individual, as well as to teach social skills (144). There now exists support for the use of CRAFT in the treatment of various addictive behaviors including alcohol, cocaine, and opioid dependence (145). The CRAFT approach has also demonstrated some support in the use of behavioral addictions (145–147). In a study of 31 concerned significant others of individuals addicted to gambling, those who received a manual based on CRAFT principles reported greater reduction of gambling in their loved ones. Training in communication skills has been shown to result in increased relationship satisfaction (148) and has demonstrated some support in the treatment of addictive disorders (149). Providing support for the use of communication skills training in the treatment of addictions, Monti et al. (150) found that among problem drinkers, those who received communication skills training in conjunction with coping skills training reported greater reduction in problematic drinking up to 12 months compared to those who received a control treatment.

## Compulsivity, maladaptive perseveration of behavior

Compulsivity refers to repetitive engagement in a behavior (151). It is also termed impairment of control. Although, compulsivity shares overlap and is often confused with impulsivity, compulsivity is conceptualized to be a distinct construct from impulsivity (151). Importantly, it has been proposed that whereas impulsivity plays a prominent role in the development of addictive behaviors, compulsivity emerges overtime and maintains addictions through a cycle of negative reinforcement (152). In other words, compulsivity serves to maintain addictions through rigid patterns of coping strategies in response to negative affect.

The incentive-sensitization theory of addictions provides empirical support for the role of compulsivity in the manifestation of addictive disorders (153). According to this theory, liking, (i.e., the hedonistic aspect of addictions) and wanting (i.e., the compulsive aspect of addictions) are two separate states. Although individuals at first engage in addictive behaviors for the hedonistic aspect, over time and with repeated engagement, individuals continue to “want” to engage in the addictive behavior without “liking” it. In other words, engaging in addictive behaviors may become a compulsion that is cue-dependent, triggered by certain situations, people, places or internal states. The incentive-sensitization theory has been applied to substance use disorders (154) as well as with behavioral addictions (153). Support for the incentive-sensitization theory comes from attentional bias research, in which problematic engagement with addictive behaviors is associated with a preferential view toward addiction-related stimulus to substances such as alcohol (155) and behaviors such as gambling (156), video games (157), food (158), and shopping (159).

Stimulus control, attentional bias retraining, and contingency management may represent potential intervention possibilities for this component vulnerability. These approaches may prevent the activation of the sensitized networks that mediate the motivation processes in compulsively engaging in the addictive behavior (154). Stimulus control is based on the principle of classical and operant conditioning and helps individuals avoid or reduce the learned association between addiction-related cues and the desire to engage in the addictive behavior. For example, stimulus control may involve avoiding certain places, people or things that have become associated with the addictive behavior. Stimulus control has been shown to be a very frequently used change strategy in recovery from addictions (160) and case studies have demonstrated the potential for the use of stimulus control in the treatment of addictions (161).

Attentional bias retraining is also another potential treatment possibility. Attentional bias refers to an unconscious process by which addicted individuals attend to addiction related cues, and subsequently have difficulties disengaging with the cues, which is thought to increase cravings and the risk of use (162). There have now been several meta-analyses that support the use of attentional bias modification in the treatment of addictive behaviors, which have demonstrated significant improvements in reducing attentional bias (162) Although the effects of attentional bias training on decreasing cravings remains unsupported, attentional bias training has demonstrated improved treatment related outcomes in problem drinkers including decreased length of stay in treatment as well as delaying the onset of relapse (163).

Contingency management is based on the principles of reinforcement and provides people tangible rewards (e.g., gift cards) for evidence of behavioral change, for example maintaining abstinence. There now exists several treatment studies supporting the use of contingency management in the treatment of a wide variety of addictive disorders, including alcohol, gambling, stimulant use, cannabis, nicotine, and opioids (164). The improved treatment outcomes not only include increased retention but also a reduction of addiction-related symptoms.

## Component model of addiction treatment

The CMAT (Figure 1) is a transdiagnostic treatment in that it can be used in the treatment of both behavioral and substance addictions. It is pragmatic in that it targets component vulnerabilities that are common to both, and that has been demonstrated to be modifiable. Importantly, the CMAT is empirically grounded in that the component vulnerabilities have all been empirically shown to be important etiological and maintaining factors for addictive behaviors and can be targeted in treatment. It is a hybrid of the three broad categories of transdiagnostic treatments described by Sauer-Zavala et al. (47). It draws upon treatment models that can be universally applied to addictive and mental health disorders, such as MET and ACT. It is also modular in that remediation of any of the specific components can be emphasized based on the specific presenting needs and treatment progress of individual clients. Finally, the CMAT fits the third category of transdiagnostic treatments identified by Sauer-Zavala (47) in that the hypothesized components included in this treatment model have been found to be core mechanistic features of addictive disorders.

In our opinion, we believe that all the components below are necessary yet insufficient in and of themselves as an effective treatment for addictions. In other words, for effective treatment, all components would need to be addressed to varying degrees. The components and their related treatment interventions are also not conceptualized as independent, but rather are linked. Indeed, treatment interventions likely impact multiple vulnerabilities. In addition, we advocate that the components listed below be individualized by modifying the varying degrees of focus on each of the components. For example, while urgency, social support, and maladaptive expectancies are all important treatment components, some individuals may require greater intervention in urgency, while others may require more focus on changing maladaptive cognitions. In this way, the CMAT is flexible in nature, without changing the underlying protocol depending on the addictive behaviors. Furthermore, we believe that the CMAT can be delivered as an individual therapy and as a group treatment, specifically as part of a step-cared approach for the treatment of addictions. This is because the components do not have to be addressed sequentially in treatment. This allows the treatment to proceed by addressing each of the components delivered via a group format. Thereafter, referrals for individualized treatments can be made based on individual needs to target the specific components. Thus, the CMAT will require clinicians to be skilled in the delivery of multiple therapeutic interventions. Clinicians will also need to be flexible in adapting the intervention possibilities based on client needs in order to address the component vulnerabilities that are maintaining the addictive behavior.

The goals of treatment (i.e., harm reduction or abstinence) will likely be dependent on several factors including the preference of the client and the clinicians views of recovery. Indeed, there is currently no one agreed upon definition of recovery, and there are multiple pathways that an individual can take to overcome their addiction (165). Furthermore, whether the goal of treatment is harm reduction or abstinence may depend on whether the addictive behavior is a behavioral or substance addiction. This is because, whereas the traditional goal of treatment for substance use disorders has been abstinence based (165), such an approach may not be possible when it comes to primary rewards such as sex and food. This has led to traditional abstinence-based 12 step programs to make exceptions such as no extramarital sexual intercourse, opposed to all sexual intercourse in the case of Sexaholics Anonymous (166) and the avoidance of certain food groups in the case of Overeaters Anonymous (167). However, these approaches have led to concerns, for example restricting any sexual activity for individuals who are not married and the potential development of disordered eating caused by avoiding certain food groups. Thus, in the case of certain behavioral addictions, harm-reduction approaches may be more appropriate. Harm-reduction approaches aim to reduce the negative consequences of addictions, as well as increase an individual's well-being. Importantly, harm-reduction has been shown to be effective in the treatment of both behavioral and substance addictions (89).

## Unique differences in addiction and its potential treatment implications

In line with Shaffer et al. (39), we recognize that different expressions of addictive disorders present with unique differences that may have important treatment implications. For example, there are differences regarding physical dependency between behavioral and substance addictions. While the presence of withdrawal symptoms are well-established for substance use disorders, it is disputable in the case of behavioral addictions (168). A recent systematic review concluded that the evidence base for withdrawal symptoms in internet gaming disorder is underdeveloped (169). Furthermore, withdrawal symptoms of behavioral addictions have largely manifest as psychological symptoms such as irritability and restlessness (168), rather than physiological symptoms, although physiological symptoms of withdrawal have been observed in gambling disorder (170, 171). The debate regarding the presence of withdrawal symptoms is not limited to behavioral addictions. Until recently, the presence of withdrawal symptoms in cannabis use disorder was debated, and was only included in the DSM-5 due to accumulating evidence (1). In a similar vein, more research is needed to demonstrate the concept of tolerance and withdrawal for behavioral addictions. The presence of withdrawal symptoms is an important factor that needs to be taken into account in the treatment of addictions as they are associated with increased risk of relapse (89). As such, a greater emphasis on the management of withdrawal symptoms may be warranted for certain addictions.

There are also differences in the physical dependency of addictions. For example, heroin, cocaine and barbiturates have been identified as having the greatest physical dependency (172). Additionally, different addictions are associated with varying degrees of both personal and interpersonal harms, with alcohol having been identified as the most harmful (173). The differences in physical dependency between addictive behaviors have basic treatment implications. For example, the risk of overdose is greater for substance use disorders, such as opioids (174) whereas the risk of overdose does not apply to behavioral addictions. Physiological individual differences may also influence the development of certain addictions, including alcohol (e.g., *ALDH2* and *ADH1B*) (175). Although physical dependence has yet to be demonstrated in behavioral addictions, certain behaviors have greater potential to lead to the development of addictive behaviors. Indeed, whereas there are countless behaviors, only a handful have been proposed to lead to addiction-related symptoms, suggesting certain compulsive behaviors have greater dependency potential than others (176).

Lastly, the negative consequences vary depending on the addictive behavior, which need to be taken into treatment considerations. For instance, the risk of sexually transmitted infections are greater for intravenous drug use (39) and compulsive sexual behaviors (177), whereas financial consequences may play a more prominent role in compulsive shopping (178) and gambling disorder (179). Additionally, individuals with gambling problems may benefit from a specific focus on the “gamblers fallacy” (i.e., erroneous cognitions about the ability to control the chance of an outcome). Individuals involved with illicit drugs, may face greater legal consequences and as such may require focus on the potential legal consequences associated with their illicit substance use. It would be of benefit for clinicians to be cognizant of these important differences and tailor the treatment accordingly.

## Current and future directions

Although the CMAT is grounded in empirical research, studies are needed to test out the assumptions of the CMAT model as there is currently no data that speak to the efficacy of the CMAT. Furthermore, studies are needed to determine whether the component vulnerabilities listed represent important mechanisms that account for treatment efficacy across a range of addictive disorders. Indeed, while we found generally strong support for the intervention possibilities listed in the CMAT model for substance use disorders, more empirical evidence is needed in the treatment of behavioral addictions, specifically other than gambling disorder. It is our hope that the model inspires both basic and applied research on these issues. Furthermore, there are likely other component vulnerabilities that have yet to be elucidated and may represent important mechanisms which can be targeted in treatment. To this end, we are currently engaging in a program of research that aims to identify and provide further empirical support for the components in the CMAT model through a multi-method approach with diverse populations. For example, we are currently conducting a quantitative study using a lay-epidemiological approach to identify the most important symptoms for 10 addictive behaviors (e.g., alcohol, cannabis, gambling, video games, sex, etc.) from people with lived experiences to identify commonalities as well as unique manifestations. Furthermore, we are assessing common clinical processes (e.g., impulsivity-compulsivity) that may be important across people seeking treatment for a variety of addictive disorders including both behavioral and substance addictions.

In regard to the CMAT, we are in the midst of developing a treatment protocol and will be testing the effectiveness of the treatment model and whether improvements are mediated by the component vulnerabilities on an individual basis, as well as a treatment protocol that will be delivered in a group format in Canada. Furthermore, we will be piloting the treatment protocol in Brazil to test whether the treatment model can be applied across diverse cultures. Future directions will involve creating an assessment tool that will have clinical validity in helping treatment providers determine which component vulnerabilities are the most important to target in treatment. Additionally, we have begun a program of research that also aims to address the treatment needs of co-occurring addictions and mental health concerns. Indeed, concurrent disorders tend to be the rule rather than the exception in addictions treatment (6). Importantly, similar component vulnerabilities have been implicated in the etiology and maintenance of mental health disorders including negative urgency (56) and impulsivity (39). To this end, we have assessed whether similar component vulnerabilities represent common factors that exacerbate the severity of mental health and addictive disorders. For example, we have found that heightened levels of impulsivity mediate the relationship between dual diagnosis of gambling and psychosis, and increased gambling severity (180). Relatedly, we have found that maladaptive expectancies mediate the relationship between co-morbid gambling and depression, and increased gambling severity. We are extending this line of work with non-treatment seeking samples as well as examining component vulnerabilities that are important in the co-morbid expression of mental health disorders and other behavioral and substance use addictions.

While we remain cautiously optimistic about the potential benefits of the CMAT, we would like to note where alternate treatment approaches may be more appropriate. First, is in the treatment of opioid dependence, which often involve the use of pharmacological treatments such as opioid agonists. In a review assessing the effectiveness of the addition of psychosocial intervention along with opioid agonists, the inclusion of psychosocial interventions did not lead to improved treatment outcomes including treatment retention, adherence to treatment or abstinence from opioid use (181). Further, the authors found that these null-results held regardless of the type of therapy intervention. In our review of the literature on the component vulnerabilities, we also found some evidence to suggest that the use of psychosocial interventions such as cue-exposure may have deleterious effects on the treatment of opioid dependence (182).

Secondly, one of the hypothesized benefits of the CMAT is in the treatment of co-morbid addictions. However, we should note that both behavioral and substance use addictions are also highly co-morbid with other mental health disorders, with high prevalence rates of co-morbid mood and anxiety disorders (54, 183). While we believe that several of the components listed in our CMAT model may be applicable to co-occurring substance use and mental health concerns, our literature review was limited to component vulnerabilities implicated across addictive disorders, as opposed to component vulnerabilities in co-occurring mental health and addictions. Thus, caution is warranted in applying our model to co-occurring addictions and mental health concerns, and we advise the use of concurrent disorder treatments in these instances.

The current and future directions noted above are only the start of an ongoing program of research. To the extent that new evidence emerges identifying new component vulnerabilities, and advancements are made in the treatment of addictive disorders, the CMAT will be revised to reflect the latest evidence base in the treatment of addictive disorders. Indeed, it is our hope through an ongoing process that the CMAT will represent an evidenced-based treatment for both behavioral and substance addictions, including addictive disorders that are well recognized, as well as emerging addictive disorders.

## Conclusion

Addictive disorders represent one of the most common psychiatric disorders in the general population and are associated with significant degradation in psychological, physical, and social impairments (184). The treatment of addictions has advanced significantly in the past several decades, with the development of evidence-based treatments (44). However, the recent proliferation of behavioral addictions has created the need for the development of a unified treatment for addictive disorders, which may help to increase the efficiency, effectiveness, and accessibility of addictions treatment for traditional and emerging addictions. The CMAT represents to our knowledge, the first attempt in developing a unified treatment approach to addictions. It is our hope that the presentation of the CMAT will generate further research in transdiagnostic mechanisms across addictive disorders and in turn, facilitate the creation of a unified treatment of addictions that may help people live a life free from their addiction.

## Author contributions

HK wrote the first draft of the manuscript. DH wrote parts of the manuscript and edited subsequent versions.

### Conflict of interest statement

The authors declare that the research was conducted in the absence of any commercial or financial relationships that could be construed as a potential conflict of interest.
